# MMP-8, TRAP-5, and OPG Levels in GCF Diagnostic Potential to Discriminate between Healthy Patients’, Mild and Severe Periodontitis Sites

**DOI:** 10.3390/biom10111500

**Published:** 2020-10-30

**Authors:** Marcela Hernández, Mauricio Baeza, Johanna Contreras, Timo Sorsa, Taina Tervahartiala, Macarena Valdés, Alejandra Chaparro, Patricia Hernández-Ríos

**Affiliations:** 1 Laboratory of Periodontal Biology, Faculty of Dentistry, University of Chile, Santiago 8380544, Chile; mhernandezrios@gmail.com; 2 Department of Pathology and Oral Medicine, Faculty of Dentistry, University of Chile, Santiago 8380544, Chile; 3 Department of Conservative Dentistry, Faculty of Dentistry, University of Chile, Santiago 8380544, Chile; mbaeza.paredes@odontologia.uchile.cl (M.B.); jcontreras@odontologia.uchile.cl (J.C.); 4 School of Public Health, Faculty of Medicine, University of Chile, Santiago 7510040, Chile; macavaldes@ug.uchile.cl; 5 Department of Oral and Maxillofacial Diseases, Helsinki University and University Hospital, 00290 Helsinki, Finland; timo.sorsa@helsinki.fi (T.S.); taina.tervahartiala@helsinki.fi (T.T.); 6 Department of Oral Diseases, Karolinska Institutet, 14152 Huddinge, Sweden; 7 Center for Climate and Resilience Research, CR2, University of Chile, Santiago 7510040, Chile; 8 Department of Periodontology, Centro de Investigación e Innovación Biomédica (CIIB), Faculty of Dentistry, Universidad de Los Andes, Santiago 7620001, Chile; chaparro.ale@gmail.com

**Keywords:** periodontitis, matrix metalloproteinase-8, tartrate-resistant acid phosphatase, osteoprotegerin, gingival crevicular fluid, biomarkers

## Abstract

Biomarkers represent promising aids in periodontitis, host-mediate diseases of the tooth-supporting tissues. We assessed the diagnostic potential of matrix metalloproteinase-8 (MMP-8), tartrate-resistant acid phosphatase-5 (TRAP-5), and osteoprotegerin (OPG) to discriminate between healthy patients’, mild and severe periodontitis sites. Thirty-one otherwise healthy volunteers with and without periodontal disease were enrolled at the Faculty of Dentistry, University of Chile. Periodontal parameters were examined and gingival crevicular fluid was sampled from mild periodontitis sites (M; *n* = 42), severe periodontitis sites (S; *n* = 59), and healthy volunteer sites (H; *n* = 30). TRAP-5 and OPG were determined by commercial multiplex assay and MMP-8 by the immunofluorometric (IFMA) method. STATA software was used. All biomarkers showed a good discrimination performance. MMP-8 had the overall best performance in regression models and Receiver Operating Characteristic (ROC) curves, with high discrimination of healthy from periodontitis sites (area under the curve (AUC) = 0.901). OPG showed a very high diagnostic precision (AUC ≥ 0.95) to identify severe periodontitis sites (S versus H + M), while TRAP-5 identified both healthy and severe sites. As conclusions, MMP-8, TRAP-5, and OPG present a high precision potential in the identification of periodontal disease destruction, with MMP-8 as the most accurate diagnostic biomarker.

## 1. Introduction

Periodontitis is a group of microbial-induced diseases characterized by gingival inflammation and host-mediated destruction of the tooth supporting tissues. The pathophysiology of the disease involves the activation of immune–inflammatory responses that trigger periodontal attachment loss and marginal alveolar bone destruction that can end up, in severe cases, in tooth mobility and final tooth loss, as well as higher risk of non-communicable diseases [1,2]. Periodontitis constitutes a global health problem that affects millions of people around the globe and requires, due to its chronic nature, continuous and lifelong monitoring and care [3].

Despite the current knowledge of periodontal diseases’ etiology and therapy [4,5], current diagnostic tools are mainly restricted to clinical and radiographic findings that represent past events of the disease (bleeding on probing, probing depth, clinical attachment loss, and radiographic bone loss) [6], and do not always exert the ability to detect the disease in its most incipient forms. Lately, periodontal research has focused on finding effective diagnostic molecular biomarkers to assist clinicians in the risk-assessment and decision-making process, as the adjustment of the therapy to the patients’ individual needs. Moreover, the new classification of periodontal diseases opened up to the future incorporation of biomarkers to aid in the case definition and classification of periodontal diseases by severity and complexity (“stage”) and risk of progression (“grade”), especially in the early detection of initial forms of the disease [7].

Gingival crevicular fluid (GFC) is a non-invasive and easily obtainable serum exudate in periodontal inflammation, which is able to carry the site-specific biologic biomarkers from the surrounding microcirculation. Evidence shows that GCF molecular constituents include markers of destruction or inflammation that may help to discriminate between healthy and diseased periodontitis sites, progression sites, or even periodontitis severity levels [8,9,10,11]. Higher degrees of sensitivity have been reached by combining different sets of GFC biomarkers; however, until now, there is no single or multiple validated biomarker for periodontal disease detection, assessment or classification [12,13].

The regulation of the delicate bone balance involves the interplay of soluble mediators including receptor activator of nuclear factor kappa-Β ligand (RANK-L), its physiologic inhibitor osteoprotegerin (OPG), and tartrate-resistant acid phosphatase-5 (TRAP-5). RANK-L is an osteolytic cytokine that binds to RANK (receptor activator of nuclear factor kappa-Β) on the mononuclear precursors. Once differentiated, activated osteoclasts express and secrete TRAP-5, accounting for bone resorptive activity. OPG is a decoy receptor that prevents RANK-L binding to RANK exerting an osteoprotective effect [14]. The neutrophil collagenase MMP-8, has been particularly studied and involved in periodontal tissue destruction, due to its particular property to degrade type-I collagen, the main constituent of periodontal extracellular matrix [10,15].

MMP-8 levels in GCF have demonstrated a high potential to identify individuals with periodontitis and reflect its severity [8,16,17], while OPG levels have been associated with the quantity and severity of periodontal bone destruction [18]. Despite its potential to reflect osteoclastic activity, TRAP-5 has been addressed in periodontitis just by few studies, supporting its potential to identify periodontitis forms in GCF and tissue samples [9,19]. In spite of the previous studies, more evidence is needed to determine the potential of these biomarkers in periodontitis, particularly in the timely detection of incipient forms and sites of disease, and their potential usefulness, hereafter, in the new classification of periodontal diseases.

The aim of this study was to assess whether MMP-8, TRAP-5, and OPG levels in GCF have diagnostic potential to discriminate between healthy patients’ mild and severe periodontitis sites. We propose that these biomarkers have a high diagnostic performance to differentiate between incipient and more advanced levels of the disease.

## 2. Materials and Methods

### 2.1. Study Population and Clinical Assessments

In this cross-sectional clinical study, periodontitis individuals (*n* = 18) and healthy volunteers (*n* = 13) from Centers of Diagnostics and Treatment of Northern Metropolitan Health Services, Santiago, Chile, were included. Inclusion criteria for periodontitis subjects encompassed a minimum of 14 natural teeth, at least three molars (excluding 3rd molars), five or more sites with periodontal probing depth (PPD) ≥ 5 mm [20], clinical attachment loss (CAL) ≥ 3 mm, and radiographic bone loss [21]. The healthy group consisted of volunteers that exhibited PPD ≤ 3 mm in every site and bleeding on probing (BOP) < 10% [22]. Exclusion criteria for all subjects were previous periodontal treatment; history of systemic disorders, such as diabetes mellitus, osteoporosis, pregnancy or nursing; or intake of medications that could affect periodontal tissues, within the past three months prior to the study. The study protocol was approved by the Ethics Committee of the Faculty of Dentistry, University of Chile, and endorsed by the FONDECYT (National Fund for Scientific and Technological Development) Bioethics Advisory Committee (FONDECYT 1090046, 28 April 2009). Procedures were undertaken with the understanding and written consent of each subject and according to ethical principles, including the World Medical Association Declaration of Helsinki.

Based on the previously-reported levels of MMP-8 [9], a minimum of 14 individuals per group was calculated to identify the differences between healthy and periodontitis groups with alpha = 0.05 and 95% power.

The subjects’ background was recorded in a medical chart that included demographic variables, current smoking, and the assessment of periodontal clinical parameters. Periodontal probing depth (PPD), clinical attachment loss (CAL), and bleeding on probing (BOP) were examined through all tooth perimeters by three calibrated examiners (JC, MB, and PH) who registered six sites in each tooth (mesiobuccal, buccal, distobuccal, distolingual, lingual, and mesiolingual) using a North Carolina manual probe (UNC-15, Hu-Friedy, Chicago, IL, USA). Sites from periodontitis patients were classified as follows: (1) initial to moderate periodontitis sites (mild sites), with CAL and PPD corresponding to those described in periodontitis stages I and II (CAL ≤ 4 mm and PPD ≤ 5 mm); and (2) severe to advanced sites (severe sites), with CAL and PPD corresponding to those described in periodontitis stages III and IV (CAL ≥ 5 mm) [7]. Healthy sites from healthy subjects (healthy sites) (PD ≤ 3 mm without BOP) [22], were also obtained. Fourteen healthy and 18 periodontitis volunteers were finally included. One individual was withdrawn from the healthy group.

### 2.2. GCF Samples

GCF samples from healthy sites from healthy individuals (H), as well as mild (M) and severe (S) periodontitis sites from diseased individuals, were consecutively collected with paper strips (Periopaper^®^, ProFlow, Amityville, NY, USA), placed into the pocket until mild resistance was sensed, and left in place for 30 s as previously reported [23]. Strips contaminated with blood or saliva were discarded. Two to six GCF samples were collected per patient. One hundred thirty two sites from 31 volunteers were sampled and frozen for the posterior immunobiochemical analyses.

GCF was eluted from the strips in a constant ratio of 80 μL of buffer containing 50 mM TrisHCl pH 7.5, 0.2 M NaCl, 5 mM CaCl_2_, and 0.01% Triton X-100 (Sigma-Aldrich, St. Louis, MO, USA), as previously reported [21].

MMP-8 levels were determined by the time-resolved immunofluorometric (IFMA) method (Hanemaaijer et al., 1997); and TRAP5 and OPG levels though a commercial Multiplex detection panel (Millipore, St. Charles, MO, USA.), according to the manufacturer’s instructions. Data were collected through a Luminex platform (Magpix, Millipore, St. Charles, MO, USA), and analyzed with the MILLIPLEX AnalystR software (ViageneTech, Carlisle, MA, USA). Results were expressed as ng/mL elution.

### 2.3. Statistical Analyses

Results were expressed at the site level as means ± standard deviation for the quantitative variables, while frequencies and proportions (%) were used for categorical variables. Group proportions were compared by Fisher’s exact test, while mean values were compared with a one-way ANOVA and Bonferroni post-hoc test. Crude and multiple linear regression models to assess the potential of biomarkers to identify healthy sites (healthy versus mild and severe periodontitis sites) and severe periodontitis sites (healthy plus mild versus severe periodontitis sites) were performed. Multiple linear models were adjusted by simultaneously entering age, gender, and smoking as covariates.

The evaluation of the diagnostic precision of the biomarkers was carried out using simple logistic regression models, with the biomarkers as explanatory variables of the clinical picture. After specifying the model, the Receiver Operating Characteristic (ROC) curves were estimated, simultaneously contrasting the sensitivity and (1- specificity) through the area under the curve (AUC) to determine the diagnostic potential of the biomarkers to identify healthy sites (healthy from mild to severe periodontitis sites), as well as their diagnostic potential to identify severe periodontitis sites (healthy plus mild from severe periodontitis sites). Additionally, this was complemented with the estimation of the Youden index, which allows establishing the optimal cut-off to achieve the highest sensitivity and specificity simultaneously and with this, the predictive capacity of the biomarker. Also, logistic regression models adjusted for age, sex, and smoking.

Statistical analyses were performed blindly, on all available data. Those observations that were under the limit of detection were imputed with the minimum value. Results were considered to be significant at the 5% critical level (*p* < 0.05). All calculations were performed using Stata V 12 package for Windows.

## 3. Results

The dataset was composed of 31 participants, of whom 13 were healthy and 18 had periodontitis. No differences were found regarding gender and smoking status between participants with periodontitis and the healthy ones (*p* > 0.05). Age in periodontitis group was significantly higher than in healthy controls (54.1 ± 8.5 versus 43.7 ± 14.0 years; *p* = 0.02) (Table 1).

Probing depths in healthy, mild, and severe periodontitis sampled sites (means ± SD) were 2.2 ± 0.40, 2.83 ± 1.20, and 6.25 ± 1.80, respectively (*p* = 0.000), while CAL measurements (means ± SD) were 1.63 ± 0.49, 2.78 ± 1.25, and 7.42 ± 2.11 in the same groups (*p* = 0.000). Healthy sites had no bleeding on probing, whereas BOP in mild and severe periodontitis sites were 71% and 83%, respectively, (Table 2).

In relation to biomarker levels in healthy and periodontitis sites according to severity (Table 2), MMP-8 had the highest levels (means) in severe periodontitis sites (464.20 ng/mL), followed by mild periodontitis sites (270.82 ng/mL), and healthy sites (60.49 ng/mL), with statistically-significant differences among all groups observed in the adjusted multilevel linear regression model (*p* ≤ 0.01). Significantly-higher levels of TRAP-5 and OPG were also found in severe periodontitis sites (478.59 and 53.3 ng/mL, respectively) in comparison to mild periodontitis sites (177.89 and 23.24 ng/mL, respectively) (*p* < 0.01), and healthy sites (56.79 and 13.12 ng/mL, respectively) (*p* < 0.01).

Table 3 shows the potential of each biomarker to identify healthy sites, discriminating from mild and severe periodontitis; and to identify severe periodontitis sites, discriminating from mild periodontitis and healthy ones. The crude (unadjusted) and the adjusted models (age, sex, and smoking) are shown. All biomarkers showed potential to identify healthy sites in the crude model (*p* < 0.05), but only MMP-8 remained significant in the adjusted model, with OPG at the limit of significance (*p* = 0.05). Additionally, all biomarkers identified severe sites, showing statistically-significant differences, for both the crude and adjusted models (*p* < 0.05).

**Figure 1:**
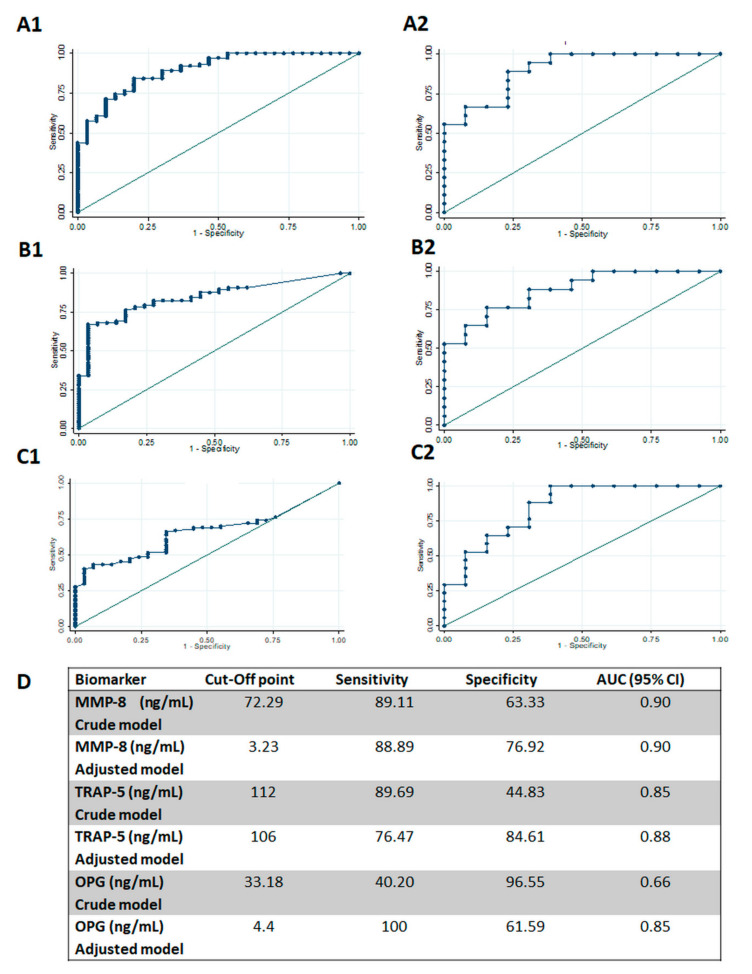
Diagnostic potential of biomarkers to identify healthy sites. (**A**). Crude (**A1**) and adjusted models (**A2**); (**B**). diagnostic potential of TRAP5 to identify healthy from periodontitis sites, crude (**B1**) and adjusted (**B2**); (**C**). diagnostic potential of OPG to identify healthy from periodontitis sites, crude (**C1**) and adjusted (**C2**); (**D**). sensitivity, specificity, and cut-off points of MMp-8, TRAP5, and OPG in their diagnostic potential to identify healthy from periodontitis sites. AUC: area under de curve. CI: confidence interval. MMP-8: matrix metalloproteinase-8, TRAP5: tartrate-resistant acid phosphatase-5, and OPG: osteoprotegerin.

**Figure 2:**
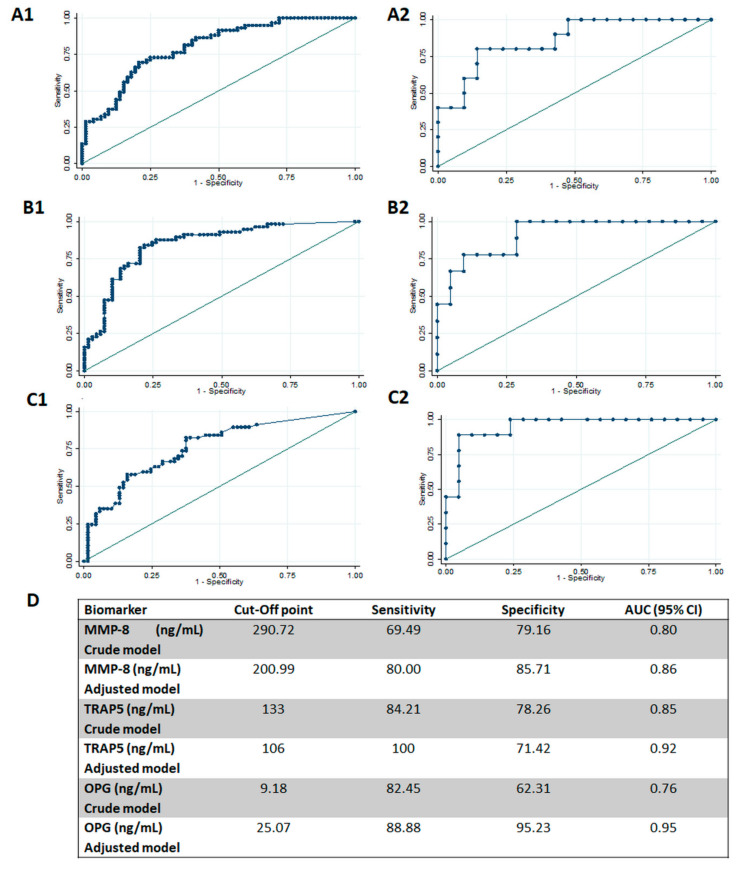
Diagnostic potential of biomarkers to identify severe periodontitis sites. (**A**). Crude (**A1**) and adjusted models (**A2**); (**B**). diagnostic potential of TRAP5 to identify healthy from periodontitis sites, crude (**B1**) and adjusted (**B2**); (**C**). diagnostic potential of OPG to identify healthy from periodontitis sites, crude (**C1**) and adjusted (**C2**); (**D**). sensitivity, specificity, and cut-off points of MMp-8, TRAP5, and OPG in their diagnostic potential to identify healthy from periodontitis sites. AUC: area under de curve. CI: confidence interval. MMP-8: matrix metalloproteinase-8, TRAP5: tartrate-resistant acid phosphatase-5, and OPG: osteoprotegerin.

Finally, the diagnostic accuracy of the studied biomarkers is illustrated with ROC curves (Figure 1 and Figure 2). ROC curves to discriminate healthy sites from mild to severe periodontitis sites showed high diagnostic accuracy for the MMP-8 crude model (AUC = 0.90, 95% CI 0.83–0.95), the MMP-8 adjusted model (AUC = 0.90, 95% CI 0.80–1.00), the TRAP-5 crude model (AUC = 0.85, 95% CI 0.77–0.91), and the TRAP-5 adjusted model (AUC = 0.88, 95% CI 0.76–0.99). A lower but still significant performance was observed for the OPG crude model (AUC = 0.65, 95% CI 0.56–0.75); however, in the adjusted model, OPG presented a high diagnostic precision (AUC = 0.85, 95% CI 0.71–0.99) (Figure 1). Optimal cut-off points to discriminate healthy sites from periodontitis sites, were obtained for each marker by using Youden’s index. The MMP-8 adjusted model showed the best performance, with a sensitivity of 89% and a specificity of 77%, at a cut-off point of 3.23 ng/mL. It was followed by the TRAP-5 adjusted model, with a sensitivity of 76% and a specificity of 85%, at a cut-off point of 106 ng/mL. Finally, the OPG adjusted model presented a sensitivity of 100% and a specificity of 62%, at a cut-off point of 4.4 ng/mL (Figure 1).

On the other hand, ROC curves of biomarkers were also designed to identify healthy plus mild sites from severe periodontitis sites (Figure 2). ROC curves showed a very high diagnostic precision, defined as AUC ≥ 0.95 for the OPG adjusted model (AUC = 0.952, 95% CI 0.88–1.00). High performance was observed for the TRAP-5 adjusted model (AUC = 0.915, 95% CI 0.81–1.00) and the MMP-8 adjusted model (AUC = 0.861, 95% CI 0.73–1.00). In the same way, optimal cut-off points were obtained for each marker by using Youden’s index. The OPG adjusted model showed the best performance, with a sensitivity of 89% and a specificity of 95%, at a cut-off point of 25.07 ng/mL. It was followed by the TRAP-5 adjusted model, with a sensitivity of 100% and a specificity of 71%, at a cut-off point of 106 ng/mL. In third place, the MMP8 adjusted model presented a sensitivity of 80% and a specificity of 86%, at a cut-off point of 200.99 ng/mL (Figure 2).

## 4. Discussion

Periodontitis impairs the supporting apparatus of the tooth, potentially leading to tooth loss, systemic alterations, and life quality disturbance [13]. Despite clinical and radiographic parameters now being the primary diagnostic methods of the disease, they mainly represent recordings of previous cumulative periodontal damage [9]. Biomarker analysis of oral fluids could provide more sensitive and timely diagnostic tools to aid in the differential diagnosis, screening, and severity determination than traditional methods, especially in susceptible individuals [13,22]. In this study, we demonstrated that MMP-8, TRAP-5, and OPG in GCF exert a high diagnostic performance in periodontitis, discriminating between incipient/early and more advanced levels of the disease.

As reported in a previous study, MMP-8, TRAP-5, and OPG in GCF showed a good discrimination performance at the site level to discriminate between healthy versus moderate to severe periodontal disease [9], especially MMP-8. This collagenase, together with MMP-9, is the main metalloproteinase in oral fluids and interstitial gingiva, with a unique ability to degrade collagen types I and III, relevant in tooth support structures [24,25]. Its association with periodontal disease has been widely reported, with a high predictive capability in the detection of periodontitis [17,26]. Pathologically-elevated levels of MMP-8 have been reported in periodontal disease in comparison to healthy patients’ sites [8,27,28], which was in accordance with this work, where MMP-8 was able to identify periodontitis sites and discriminate according to its severity. Indeed, a recent systematic review that included two studies from our research group (Leppilahti, Hernandez-Rios et al., 2014, Baeza, Garrido et al., 2016), considered MMP-8 as the most useful single biomarker to aid in the clinical diagnosis of periodontitis [26], with medians of sensitivity and specificity of 76.7% and 92%, respectively. MMP-8 was also the molecule with the best diagnostic performance in the present study, with high accuracy ROC curves that discriminated healthy versus periodontitis sites (sensitivity of 89% and a specificity of 77%), and, to a lesser amount, early versus severe periodontitis sites, in the final adjusted models. Similar to Gursoy et al., we found that MMP-8 was the main biomarker to distinguish severe from mild periodontal and healthy sites in adjusted models [24], while TRAP-5 and OPG were significant both in unadjusted and adjusted regression models to distinguish healthy plus mild sites from severe periodontitis sites. That agrees, in part, with previous studies, in which MMP-8 and OPG, in combination with anaerobic pathogens, could be able to predict an individual’s periodontal status [29]. It is noteworthy that these studies were performed utilizing diagnostic assays with different antibodies for MMP-8 [17].

OPG is an important regulator of bone resorption that alters the function and survival of mature osteoclasts. It acts locally and systemically by binding to RANK-L, blocking its interaction with RANK in order to inhibit osteoclast differentiation [14,30]. The proportion of and interaction between these molecules are important in the regulation of osteoclastogenesis and alveolar bone resorption. Even when OPG tends to be lower in periodontitis in relation to healthy individuals [14,31,32,33], oscillating concentrations of this molecule and a consistently-higher RANK-L/OPG ratio have been reported in different periodontal studies [14,32,34,35].

Tartrate-resistant acid phosphatase (TRAP-5), on the other hand, is an enzyme released by active osteoclasts, together with other bone degradation products that directly reflects bone resorption [9]. In this study, OPG and TRAP-5 discriminated healthy plus mild diseased sites from severe periodontitis sites, with lower OPG and higher TRAP-5 levels in sites with increased periodontal destruction, as could be expected [9]. OPG presented the highest diagnostic precision within biomarkers to discriminate severe periodontitis sites from sites with a lesser severity, while TRAP-5 models presented the second-highest discrimination performance in periodontitis severity, when comparing healthy versus periodontitis groups. Even when barely studied in periodontitis forms, this work supports TRAP-5 as a promising GCF biomarker of this disease [9].

Tissue inhibitors of matrix metalloproteinase (TIMPs) regulate MMPs activity, and the imbalance between both kinds of biomolecules could promote periodontal tissue destruction [13]. Negative correlations were shown between periodontal disease severity and GCF TIMP-1 and -2 levels, together with a positive correlation to MMP-8 and -9 activities [36]. Although the absence of MMP-8/TIMPs ratio calculation might represent a limitation of the current work, the literature reported opposed results for matrix metalloproteinase inhibitors for periodontal diagnosis. TIMP levels were shown decreased, enhanced, or unaltered in adult/juvenile periodontitis and after periodontal therapy [27,37,38]; while MMP-8, on the other hand, represented a consistently-promising periodontal biomarker itself [17,26,39]. Future studies should confirm TIMPs’ testing value in the diagnosis of incipient stages and grades of periodontitis.

The host inflammatory response could be affected by many local and general factors that should be considered; however, just a few studies of periodontal biomarkers as diagnostic aids consider and adjust for confounding factors. In the present work, we adjusted regression models and ROC curves by age, gender, and smoking. Age was the only covariable heterogeneously distributed in the sample, where periodontitis volunteers were older than the healthy ones. Epidemiological aspects of the disease may explain this limitation, in part. The prevalence and severity of periodontitis, measured through clinical attachment loss and missing teeth, increase linearly with aging due to by lifelong cumulative periodontal damage [40,41,42]. Wide age ranges and a lack of age homogeneity are not scarce in the literature, with mean ages of studies that varies between 31.5 and 59.2 years in a recent systematic review [39].

Smoking is a major preventable risk factor for periodontitis that alters the innate and adaptive immune system [43]. Gingival crevicular levels of MMP-8 in the literature were lower in smokers than in non-smokers. Meanwhile, higher baseline or post-treatment levels of the enzyme in smokers were associated with unstable periodontal disease and predicted weak periodontal therapy outcomes [44,45]. OPG levels in plasma and GCF decreased in smokers with periodontitis, with an increasement of RANKL/OPG ratio [46,47,48]. Systemic diseases have also been associated with biomarker fluctuation, like cardiovascular and atherosclerotic diseases in relation to metalloproteinases, and OPG, eventually, regarding post-menopausal women with osteoporosis [17,18]. In order to avoid confounding factors, individuals with history of systemic disorders, like osteoporosis and diabetes, were excluded from the present work.

The results of this study could be explained, at least in part, by the clinical characteristics of the sites. Severe sites showed significantly-different and markedly-advanced periodontal parameters in relation to mild and healthy sites. The major clinical periodontal destruction in severe sites could presumably be associated with a higher bone loss, reflecting the better performance of TRAP-5 and OPG as biomarkers.

The 2017 World Workshop classified periodontitis in stages and grades, according to the disease severity and progression risk, respectively. Its framework opened up the incorporation of future validated biomarkers to aid in early periodontal diagnosis and classification, as the actual grading system might help to predict future periodontal breakdown, but not necessarily in a timely enough manner to prevent severe damage or to provide cost-effective therapies [2,28]. Periodontitis stage is initially determined by clinical attachment loss as a severity parameter, and even when the new classification categorizes periodontal patients at an individual level by an assortment of added clinical parameters [49], it is interesting to study how the new classification behaves in relation to different sites with varying severities, in the same or different patients. So, clinical attachment loss and probing depths, as the classic clinical reference parameters for periodontal diagnosis [26], were considered in this study, applying at, a site level, the limits for stages I and II (initial and moderate periodontitis) versus stages III and IV (moderate to advanced periodontitis), called here “mild” and “severe” sites. There is almost no present study of biomarkers in relation to the new periodontal disease classification. A recent short communication by Sorsa et al. found significantly-lower levels of MMP-8 in healthy individuals, in comparison with those from upper stages and grades [28]. The report suggested that the MMP-8 mouth rinse test (based on a simple method to collect and sample GCF) might be a promising future adjunctive and preventive method in diagnosing, staging, and grading periodontitis.

Finally, it seems that molecules associated with bone and soft tissue degradation are the most promising biomarkers in periodontitis. Nevertheless, and due to the multifactorial and episodic nature of the disease (including environmental and inter- and intra-individual variations), no single or combined biomarker has been validated in periodontal disease assessment [13]. Future quality, larger, longitudinal, and multicenter studies that evaluate the performance of single and multiple biomarkers at individual and site-level are needed in order to aid in the classification, early detection, and risk assessment of tooth supporting tissue diseases.

## 5. Conclusions

MMP-8, TRAP-5, and OPG present a high precision diagnostic potential in the identification of periodontal disease destruction. While TRAP-5 and OPG perform well in discriminating severe sites, MMP-8 is the most accurate biomarker to identify different levels of periodontal health and disease.

## Figures and Tables

**Table 1:** Characteristics of study participants.

Parameter	Healthy (*n* = 13)	Periodontitis (*n* = 18)	*p*
Age	43.7 ± 14.0	54.1 ± 8.5	0.02
Gender (females)	7	12	>0.05
Smoking	2	3	>0.05

Data presented as mean ± standard deviation or absolute frequencies.

**Table 2:** Clinical parameters and levels of biomarkers in healthy, mild, and severe periodontitis sites.

Parameter	H (*n* = 30)	M (*n* = 42)	S (*n* = 59)	Overall *p*	H v/s M *p*	H v/s S *p*	M v/s S *p*
PPD (mm)	2.2 ± 0.40	2.83 ± 1.20	6.25 ± 1.80	0.000	0.187	0.000	0.000
CAL (mm)	1.63 ± 0.49	2.78 ± 1.25	7.42 ± 2.11)	0.000	0.010	0.000	0.000
BOP *n* (%)	0	30 (71%)	49 (83%)	0.000	0.000	0.000	0.346
MMP-8 (ng/mL)	60.49 ± 95.67	270.82 ± 238.96	464.2 ± 281.31	0.000	0.001	0.000	0.000
TRAP-5 (ng/mL)	56.79 ± 65.73	177.89 ± 221.46	478.59 ± 422.73	0.000	0.345	0.000	0.000
OPG (ng/mL)	13.12 ± 11.49	23.24 ± 43.18	53.3 ± 57.88	0.000	1.000	0.001	0.006

Data presented as mean ± standard deviation or absolute frequencies (%). H: healthy sites, M: mild periodontitis sites, S: severe periodontitis sites, PPD: periodontal probing depth, CAL: clinical attachment level, BOP: bleeding on probing, MMP-8: matrix metalloproteinase-8, TRAP-5: tartrate-resistant acid phosphatase-5, and OPG: osteoprotegerin. ANOVA with Bonferroni test.

**Table 3:** Linear regression models to identify biomarkers’ potential to identify healthy sites (healthy versus mild and severe periodontitis sites) and severe periodontitis sites (healthy plus mild versus severe periodontitis sites).

Healthy Sites (H v/s M and S)	Crude Model		Adjusted Model	
	OR (95% CI)	*p*-value	OR (95% CI)	*p*-value
MMP-8	1.011 (1.005–1.016)	0.000	1.009 (1.001–1.018)	0.021
Intercept	0.614 (0.316–1.192)	0.150	0.015 (0.00–2.525)	0.109
TRAP5	1.014 (1.006–1.022)	0.001	1.017 (0.996–1.039)	0.096
Intercept	0.646 (0.316–1.322)	0.232	0.013 (0.001–1.708)	0.081
OPG	1.036 (1.009–1.064)	0.009	1.072 (1.000–1.150)	0.050
Intercept	1.561 (0.866–2.812)	0.138	0.006 (0.000–0.976)	0.049
**Severe Sites** **(H and M v/s S)**	**Crude Model**		**Adjusted Model**	
	**OR (95% CI)**	***p*-value **	**OR (95% CI)**	***p*-value **
MMP-8	1.004 (1.002–1.005)	0.000	1.005 (1.000–1.010)	0.018
Intercept	0.229 (0.124–0.423)	0.000	0.010 (0.000–6.103)	0.160
TRAP5	1.005 (1.002–1.007)	0.000	1.009 (1.000–1.019)	0.039
Intercept	0.236 (0.128–0.435)	0.000	0.021 (0.000–18.763)	0.266
OPG	1.023 (1.009–1.037)	0.001	0.146 (1.020–1.287)	0.021
Intercept	0.410 (0.245–0.687)	0.001	0.000 (2.66e^−09^–8.165)	0.113

OR: odds ratio. CI: confidence interval. H: healthy sites, M: mild periodontitis sites, S: severe periodontitis sites, MMP-8: matrix metalloproteinase-8, TRAP5: tartrate-resistant acid phosphatase-5, and OPG: osteoprotegerin.
